# Criminally violent victimisation in schizophrenia spectrum disorders: the relationship to symptoms and substance abuse

**DOI:** 10.1186/1471-2458-12-445

**Published:** 2012-06-18

**Authors:** Mairead C Dolan, David Castle, Kate McGregor

**Affiliations:** 1 Centre for Forensic Behavioural Science, Monash University, 505 Hoddle Street, Clifton Hill, VIC, 3068, Australia; 2 St Vincent’s Hospital, Department of Psychiatry, University of Melbourne, 41 Victoria Parade, Fitzroy, VIC, 3065, Australia

## Abstract

**Background:**

Violent victimisation among people with major mental illness is well-documented but the risk factors for criminal violent victimisation are not well understood.

**Methods:**

We examined the relationship between illness-related variables, indices of substance abuse and previous history of violence in a sample of 23 male criminally violently victimized and 69 non-criminally violently victimized male patients with DSM-IV-TR diagnoses of schizophrenia and schizoaffective disorder that were resident in the community and in contact with public mental health services in Victoria Australia. Data on criminal victimisation was acquired from the police database.

**Results:**

Demographic, a history of violence or illness-related variables did not distinguish between those had been the victim of a violent crime and those who had not. Our data indicated that drug abuse was a key factor in distinguishing between the groups, but the age of onset of substance abuse was not a significant factor. Scores on measures of drug abuse were modest predictors of criminal victimisation status in our Receiver Operator Characteristic analyses.

**Conclusion:**

Overall, our findings suggest that substance abuse (particularly drug abuse) is a key predictor of violent victimisation based on criminal statistics. The latter has implications for mental health professions involved in the care planning and community management of patients with major mental illness and work points to the importance of substance abuse treatment in the prevention of victimisation as well as violence perpetration.

## Background

Over the last three decades there has been extensive research looking at the links between mental disorder and violence and data suggests that substance abuse, previous violence, psychopathic traits and threat control override symptoms elevates the risk of violence in mentally disordered samples [1-8]. However, there is also a growing literature to suggest that major mental disorder is associated with an increased risk of violent victimisation [9-20]. Although rates of victimisation vary across studies, work by Teplin et al. [11] based on the National Crime Victimisation Study in Chicago indicated that psychiatric patients were up to 11 times more likely to be victimized by violent crime compared to non-psychiatric patients after controlling for demographic differences.

Psychiatric patients with a history of violent or antisocial behaviour have higher rates of victimisation than those who do not [10,13-15,17,19,21-26]. Many of the psychosocial risk factors for violence in mentally disordered populations are similar to those for violent victimisation including; younger age, homelessness, socioeconomic disadvantage, active symptoms of mental illness, personality disorder, and substance abuse [13,22,24,27-29].

There are a number of studies suggesting links between victimisation and a range of illness related variables include active symptoms [15,21,23,26,30], a younger age of illness onset [15,21,22,27], and a chronic course of illness [14]. Substance abuse has also been linked with violent victimisation among mentally disordered individuals [9,14,16,22-24,27,28,30].

In this study, we examined the nature of relationships between demographic variables, illness-related variables including current symptoms, prior history of violence, and substance abuse- related factors in a cohort of patients with schizophrenia spectrum disorders who were resident in the community and who had or had not been victims of violent crime according to official criminal databases. This is the first cohort study in Australia to have examined the nature of the relationship between symptoms, substance abuse and violent victimisation.

As we recruited a clinically stable sample of patients with major mental illness, we postulated that a history of violence, and substance abuse would be more significant predictors of violent victimisation than symptoms of mental illness

## Methods

### Participants

Ninety-four male participants, aged 18–65, who met DSM-IV-TR [31] criteria for schizophrenia, schizophreniform disorder or schizoaffective disorder, were recruited from six Victorian community mental health clinics in the Melbourne metropolitan area. One hundred and seventeen outpatients meeting inclusion criteria for the study were approached to participate in the present study after being referred by their treating clinicians. Ninety seven participants consented to participate in the study. Of these, three participants were excluded: one due to being deemed not to have capacity to provide informed consent; one due to refusing to consent to release any collateral information for the research project (neither psychiatric case file nor police record); and one due to not meeting DSM-IV-TR (APA, 2000) criteria for a schizophrenia-spectrum disorder on review. Two participants refused consent to access their Victoria Police files, but participated in all other aspects of the research and so were included in analysis. No participants withdrew from the study.

We focused on a male only sample as there were insufficient females to permit separate analyses and females are likely to have had different victimisation experiences. In order to ensure cases met criteria for inclusion, medical files were reviewed and checked to ensure they met DSM-IV criteria. Exclusion criteria were; inability to give informed consent, clinically unstable and significant head injury. The mean age of the sample was 35.06 years (*SD* = 8.05; range 19–61 years). The majority (70.2%) was of Anglo-Saxon ethnicity, with the remaining participants of European, Pacific Islander, Asian or African ethnicity. The mean age of illness onset was 23.24 years (*SD* = 5.87) and the mean duration of illness 11.82 (*SD* = 8.25) years.

### Procedure and measures

The study was approved by Monash University, St Vincent’s Hospital, Barwon Health, Eastern Health, Peninsula Health, Bendigo Health and, Victoria Police Human Research Ethics Committees. Written informed consent was obtained from all participants. Interviews were conducted over one to two sessions as required and all self-report measures were completed via interview to ensure good comprehension and overcome potential literacy problems. Victim of crime information for each consenting participant (*N* = 92) was extracted from the Victoria Police Law Enforcement Assistance Program (LEAP) criminal database which stores and retains all particulars of all crimes brought to the notice of police in the state of Victoria, Australia. The LEAP *Alleged Victim* database provides data on victims and offenders (if known) of all alleged crimes whether or not this results in apprehension of the offender by Victoria Police. This resulted in 23 cases where the participants had been the Victim of a Violent Offence (VVO) and 69 participants who had not been the Victim of Violent Offence (NVVO). The VVO cases referred to cases of individuals who had been the victim of offences classified within the *Assault, Homicide,* and *Armed Robbery* categories of offences created by Victoria Police. As this study focused on physical violence, *Threat to Kill* charges were not classified as violent offenses for our purposes.

### Assessment of symptoms

Schizophrenia symptoms were assessed using the interview based Positive Symptom and Negative Symptom subscales from the Very Brief Psychosis Treatment Scale (VBPTS [32]. The VBPTS was designed as a brief alternative to the full Positive And Negative Symptom Scale (PANSS) [33]: it covers both positive and negative symptoms using PANSS-derived anchors, such that higher scores reflect higher symptom ratings. The mean score on the VBPTS- Positive Symptom Scale was 6.17 (*SD* = 3.40) out of a possible 21. The mean VBPTS Negative Symptom Scale score was 5.93 (*SD* = 3.24) out of a possible score of 21. These scores suggest a low level of current psychotic symptomatology.

### Assessment of substance abuse

Participants were administered the Drug Abuse Screening Test (DAST), a 28-item self-report measure of problematic substance use [34]. Questions were adapted to assess lifetime use as it has been shown to be a valid measure of lifetime drug abuse in psychiatric populations [35]. The DAST is scored in a binary (yes/no) format except for three reverse-keyed items. Item response scores are summed to produce a total score ranging from 0 to 28, with higher scores indicating an increased presence of symptoms of substance abuse. A cut-off score of 6 is generally used to indicate a drug abuse or dependence problem [36]. For the present study DAST scores were used as a continuous and categorical measure. The mean DAST score was 8.71 (*SD* = 7.3). Two participants refused to complete drug use measures and these cases were excluded from the analysis. In addition to DAST scores, data was collected on self-reported age of onset of regular alcohol and illicit drug use, and lifetime poly-substance use. Thirteen participants reported no previous history of illicit drug use and so these cases were excluded from analysis of age of drug use onset. Polysubstance use was defined in this instance as having a history of using three or more illicit substances at a frequency of monthly or greater. This category was developed from the DSM-IV-TR [31] guidelines which define polysubstance use in the *polysubstance dependence* diagnosis as using three or more illicit substances. Criminal records were also examined for apprehensions for drug-related offences.

In addition, self-report information was gathered to reflect participants’ frequency and quantity of alcohol use over their lifetime. This information was gathered during the interview process via self-report. This information was used to create two dichotomous variables measuring whether participants had a lifetime history of daily alcohol use, or a lifetime history of a harmful alcohol use pattern. Harmful alcohol use was defined as a typical pattern of consuming more than four standard drinks per setting. This category was created based on the National Health and Medical Research Council (NHMRC, 2009) *Australian Guidelines to Reduce Health Risks from Drinking Alcohol*, in which Guideline 2 states that: “for healthy men and women, drinking no more than four standard drinks on a single occasion reduces the risk of alcohol-related injury arising from that occasion” (p. 51).

### Assessment of previous history of violence

Lifetime violent offending (from age 18 years) was measured dichotomously utilising the Victoria Police Law Enforcement Assistance Program (LEAP) criminal database. The LEAP *Alleged Offender* database provides data on all offenders and victims of all alleged crimes which resulted in any contact with Victoria Police from 1993 onwards. Violent offences were considered as those classified within the *Assault, Homicide,* and *Armed Robbery* categories of offences created by Victoria Police. Consistent with the definition of violence used in the creation of victimisation groups, *Threat to Kill* charges were not classified as violent offenses for our purposes. Consent for access to LEAP files was not granted in 2 cases so these were excluded from this aspect of the analysis.

### Statistical analysis

Data were analysed using the Statistical Package for the Social Sciences (SPSS) version 17.0 Chicago Illinois. Comparisons between those who were victims of a violent offence (VVO) and those who were not victims of a violence offence (NVVO) were conducted using chi squared analyses and independent t tests as appropriate. Receiver Operating Characteristics (ROC) Area Under the Curve (AUC) statistics [37] were used to examine the predictive key dimensional variables and violent victimisation group status with an AUC of 0.50 representing chance and AUCs >0.75 as moderate to good.

## Results

### Demographic data

There were no significant group differences on demographic variables between those who had been the victim of a violent offence (VVO) and those who had not (NVVO). See Tables 1 and 2.

**Table 1:** Demographic and background characteristics for the No Victim Violent Offence and Victim Violent Offence groups

	**No Victim Violent Offence (NVVO) (*N* = 69)**		**Victim Violent Offence (VVO) (*N* = 23)**				
	**Mean**	***SD***	**Mean**	***SD***	***df***	***t***	***p***
Age	35.39	8.16	34.35	8.10	90	.53	.596
Years of Education	11.72	1.86	11.04	1.30	90	1.63	.108
Age at first psychosis diagnosis	23.09	5.27	24.13	7.41	29.76	−.620	.540
Years since first psychosis diagnosis	12.30	8.67	10.22	7.19	90	1.04	.303
Percent of life with psychosis diagnosis	32.17	18.09	28.86	16.51	90	.778	.439
**DAST**	**7.40**	**6.99**	**11.91**	**7.48**	**88**	**−2.62**	**.010**
Age of alcohol use onset	15.41	3.23	14.00	2.95	86	1.87	.066
Age of drug use onset	16.24	3.16	16.57	5.95	24.22	−.32	.811

*Note.* NVVO = No Victim Violent offence; VVO = Victim of Violent Offence. DAST = Drug Abuse Screening Test.

**Table 2:** Demographic and background characteristics for dichotomous variable in No Victim Violent Offence and Victim Violent Offence groups

	**NVVO (*N* = 69)**	**VVO (*N* = 23)**	***x*²**	***p***
Born in Australia	59 (85.5%)	23 (100%)	.030	1.000
Single	56 (81.2%)	19 (82.6%)	.024	1.000
Unemployed	46 (66.7%)	20 (87.0%)	3.503	.061
**DAST – drug use problem-** total score ≥ 6	**33 (47.8%)**	**17 (73.9%)**	**4.22**	**.040**
**Apprehension for drug offence**	**9 (13.0%)**	**11 (47.8%)**	**12.27**	**<.001**
**Lifetime polysubstance abuse**	**11 (16%)**	**15 (65.2%)**	**20.25**	**<.001**
Lifetime history of harmful alcohol use pattern	59 (85.5%)	22 (95.7%)	1.39	.239
Lifetime history of daily alcohol use	32 (46.3%)	13 (56.5%)	.62	.431

*Note.* NVVO = No Victim Violent offence; VVO Victim of Violent Offence; DAST = Drug Abuse Screening Test.

### Substance abuse

There were higher mean DAST scores in the VVO group than the NVVO group and a higher proportion of participants in the VVO group scored above the DAST cut-off suggestive of a drug use problem. A significantly higher proportion of the VVO group had perpetrated a drug-related offence and had a history of polysubstance abuse than those in the NVVO group. There were no significant group differences with respect to the proportion of the groups with a lifetime history of daily or harmful alcohol use and there were no significant group differences with respect to the mean age of drug or alcohol use onset (see Tables 1 and 2). DAST scores were predictive of victim of violent offence group membership at a level that was modestly significantly above chance [AUC = .680, *SE* = .066, 95%CI (.551, .809), *p* = .010].

### Symptomatology

There were no significant group differences for the mean scores on the VBPTS Positive or Negative Symptom scales or any of the scale items (see Table 3). Neither the Positive nor Negative VBPTS subscales performed above chance in predicting victim of violent offence group membership (Positive subscale: AUC = .423, *SE* = .068, 95% CI [.291, .556], *p* = .273; Negative subscale: AUC = .468, *SE* = .080, 95%CI [.311, .624], *p* = .642).

**Table 3:** Mean VBPTS positive and negative symptom scale scores for the No Victim Violent Offence and Victim Violent Offence groups

	**NVVO (*N* = 69)**		**VVO (*N* = 23)**				
	**Mean**	***SD***	**Mean**	***SD***	***df***	***T***	***p***
Positive symptoms	6.39	3.44	5.57	3.42	90	1.10	.27
Delusions	2.17	1.52	1.91	1.47	90	.72	.475
Hallucinations	2.28	1.61	2.09	1.65	90	.48	.630
Suspiciousness	2.00	1.35	1.57	.99	51.24	1.65	.105
Negative symptoms	5.74	2.70	6.35	4.41	29.58	.002	.99
Blunted Affect	2.15	1.23	2.39	1.78	29.32	−.62	.542
Lack Spontaneity Conversation	1.55	.93	2.00	1.51	27.82	−1.35	.189
Passive Social Withdrawal	2.06	1.25	1.87	1.14	90	.64	.524

*Note.* VBPTS = Very Brief Psychosis Treatment Scale; NVVO No Victim of Violent offence; VVO = Victim Violent Offence.

### Violence

There were no significant group differences in the proportion of VVO versus NVVO participants with a history of a violent offence (VVO: 39.1%, *n* = 9 vs. NVVO: 26.1%, *n* = 18), *χ*² = 1.415, *df* = 1, *p* = .234).

## Discussion

In this study, we examined the relationship between symptoms, indices of substance abuse, prior history of violence and criminally violent victimisation in a cohort of male patients with schizophrenia spectrum disorders who were resident in the community in six Victorian public mental health services. We did not include females in the sample as we there are gender differences in the nature of victimisation [11] and we were interested in violent victimisation which is seen more frequently in men [11]. In this cohort 25% of the sample had been a victim of a violent offence. The latter figure is similar to data from the UK where 23% were victims of violent crime [15]. A systematic review of the literature suggested criminal victimisation rates (based on self report) range from 4.3%–35% depending on the time period understudy [38].

In our comparison of those who were victims of violent crime and those who were not based on the crime database, we found no significant group differences in the demographic characteristics of the samples. Previous studies have found an association between unemployment and victimisation [13,23,24]. Given that the unemployment was approaching significance it is likely this factor would have reached statistical significance with a larger sample size. Studies looking at the relationship between age and violent victimisation have produced mixed findings. While there are reports that young age is a risk factor for victimisation [e.g. [15,21,22,24,27], we did not find this to be the case and our findings fit with other reports that young age may not be robustly associated with victimisation [e.g. [14,26]. Differences between the samples under study and the lifetime measure of victimisation may, however, account for the divergent findings.

Unlike previous studies [e.g. [15,21,26,27], we did not find a robustly significant relationship between early illness onset and violent victimisation. Honkonen et al. [22] reported that a shorter duration of illness was associated with increased risk for violent victimisation within a three year period. Differences in the timeframes in which violent victimisation is examined may account for the divergent findings.

In this sample we did not find any significant relationship between symptoms and violent victimisation status. It has been suggested that the association between positive symptoms and criminally violent victimisation is more apparent in the acute phase of illness when patients may present as either vulnerable or behaviourally disturbed during their community interactions [26]. The latter notion is consistent with some [14,22], but not all previous studies [15,21,23,30]. It is likely that the divergent findings in the literature may reflect differences in the nature of the samples studied, methodology, and the timeframe in which violent victimisation was examined. In this study we looked at lifetime victimisation in a cohort of clinically stable patients making it difficult to draw definitive conclusions on the role of the acute phase of illness in their histories of victimisation.

In line with our *a priori* hypothesis, we found significant associations between substance abuse and victimisation, with significantly higher DAST scores in the violently victimized group. In particular, we found that lifetime poly-substance abuse was significantly more prevalent in those who were victims of violent crime, as was an arrest for a drug related offence. In this study, the mean age of onset for drug or alcohol use did not differ significantly between those who were and those who were not victims of violent crime. We would have thought that the latter may be a significant factor as the early onset of substance abuse tends to be associated with antisocial personality pathology which is in turn is known to be a risk factor for both violence and violent victimisation [15,21]. As this is the first study to investigate a relationship between age of substance use onset and violent victimisation, this is an area that warrants further study before any definitive conclusions can be drawn on the significance of early onset substance in the victimisation literature. Overall our findings largely fit with previous reports that substance abuse problems are a risk factor for victimisation in those with major mental disorders such as schizophrenia [9,12,14,15,22,23,27,30]. Although previous studies have found an association between alcohol abuse and victimisation [e.g. [9,22,23,27], we did not find that this was the case in this sample. It is possible that the high rates of alcohol abuse problems in both groups may have masked any significant findings. It may also reflect differences in alcohol use measurement between studies. Alcohol abuse has been recognised as a significant correlate of violent victimisation in a recent systematic review [38] and we would have anticipated that it would be a significant correlate of violent victimisation. It is possible that a more in-depth measure of the extent of alcohol abuse may have resulted in more notable difference between groups. Further work is needed to clarify the specificity of different types of substance abuse problems in the violent victimisation literature.

In this study, we found no association between violent crime victimisation and perpetration. This is not consistent with previous studies documenting an association between violence and violent victimisation in patients with major mental disorder [10,12,13,17,19,22-25,29]. Further work is therefore needed to explore potential differences in the proximal and distal factors that mediate the relationships between violence and violent victimisation in general populations and clinical samples if we are to gain a better understanding of how victim-perpetrator conflicts and interactions emerge as predominately one or the other.

### Strengths and limitations

This is the first study looking at the correlates of victimisation in terms of demography, substance abuse, illness related variables and previous violence in a community sample of Australian patients with major mental illness; however, we acknowledge that there are some strengths and limitations that need to be outlined.

We used official databases as the means of assessing both violence and violent victimisation. This may have resulted in an underestimation of true violence and victimisation rates as these incidents may not be reported to police. However, there are also problems in relying on self-report due to recall bias so future work should use a combination of sources.

The study sample size was relatively small and confined to males. The findings can therefore not be extrapolated to females or mixed gender samples where gender differences in types of victimization may be apparent. This was a cross sectional study that collected data on lifetime rather than prospective post interview victimisation which makes it impossible to draw any firm conclusions on causal relationships but may also account for the lack of a significant relationship between victimisation and current symptoms. It is possible that the inclusion of patients with a greater variance in symptom scores may have resulted in the emergence of significant differences between those who were and were not victimised, but ethics approvals required the inclusion of those able to give valid consent. Prospective follow studies are needed to explore the context in which victimisation takes place in order to gain a more in-depth understanding of the nature of the victim-offender relationship and the role of active symptoms. This study adds to the evidence base that symptoms of mental illness and co-morbid substance abuse, as well as individual differences in violence potential may be important factors in victimisation, but it is important to note that there have been no notable developments in the provision of comprehensive services that also minimise risk of victimisation.

## Conclusions

Overall, the present study suggests that substance abuse is a key factor in predicting violent criminal victimisation. Whether the link between substance abuse and victimisation is medicated by underlying personality pathology warrants further investigation. As substance abuse is also a key factor in the prediction of future violence in those with major mental illness [2,39,40], this is an important treatment target for clinicians working with individuals with major mental illness such as schizophrenia in a range of mental health settings, but particularly the community where risk of adverse outcome may be higher.

## Abbreviations

NVVO: No victim violent offence; VVO: Victim of violent offence; VBPTS: Very brief psychosis treatment scale; DAST: Drug abuse screening test; ROC: Receiver operating characteristic; AUC: Area under the curve; LEAP: Law enforcement assistance program.

## Competing interests

The authors declare they have no competing interests.

## Authors’ contributions

MD led the overall study and was primary supervisor to KMcG who conducted the field data collection. DC was co-supervisor and assisted with data analysis and interpretation. All authors were involved in data analysis and write up and have read and approved the final manuscript.

## Pre-publication history

The pre-publication history for this paper can be accessed here:

http://www.biomedcentral.com/1471-2458/12/445/prepub

## References

[B1] Link BG Stueve A Monahan J, Steadman HJ Psychotic symptoms and the violent/illegal behaviour of mental patients compared to community controls Violence and mental disorder: Developments in risk assessment 1994 University of Chicago Press, Chicago 137 159

[B2] Steadman HJ Mulvey EP Monahan J Clark P Appelbaum PS Grisso T Silver E Violence by people discharged from acute psychiatric inpatient facilities and by others in the same neighborhoods Arch Gen Psychiatry 1998 55 393 401 10.1001/archpsyc.55.5.393 9596041

[B3] Skeem J Monahan J Mulvey EP Psychopathy, treatment involvement, and subsequent violence among civil psychiatric patients Law Hum Behav 2002 26 577 603 12508696 10.1023/a:1020993916404

[B4] Monahan J Steadman HJ Silver E Appelbaum PS Clark Robbins P Mulvey EP Banks S Rethinking risk assessment: The MacArthur study of mental disorder and violence 2001 Oxford University Press, New York

[B5] Doyle M Dolan M Standardized risk assessment Psychiatry 2007 6 10 409 414 10.1016/j.mppsy.2007.07.004

[B6] Dolan M Doyle M Violence risk prediction: Clinical and actuarial measures and the role of the Psychopathy Checklist Br J Psychiatry 2000 177 303 311 10.1192/bjp.177.4.303 11116770

[B7] Dolan M Doyle M Violence risk assessment: Combining actuarial and clinical information to structure clinical judgements for the formulation and management of risk J Psychiatr Ment Health Nurs 2002 9 6 649 658 10.1046/j.1365-2850.2002.00535.x 12472817

[B8] Doyle M Shaw J Carter S Dolan M Investigating the Validity of the Classification of Violence Risk in a UK Sample Int J Forensic Ment Health 2010 9 4 316 323 10.1080/14999013.2010.527428

[B9] Hiday VA Swartz MS Swanson JW Borum R Wagner HR Criminal victimisation of persons with severe mental illness Psychiatr Serv 1999 50 62 68 9890581 10.1176/ps.50.1.62

[B10] Hiday VA Swanson JW Swartz MS Borum R Wagner HR Victimisation: A link between mental illness and violence? Int J Law Psychiatry 2001 24 559 572 10.1016/S0160-2527(01)00091-7 11795220

[B11] Teplin LA McClelland GM Abram KM Weiner DA Crime victimisation in adults with severe mental illness: Comparison with the national crime victimisation survey Arch Gen Psychiatry 2005 62 911 921 10.1001/archpsyc.62.8.911 16061769 PMC1389236

[B12] Silver E Arseneault L Langley J Caspi A Moffitt TE Mental disorder and violent victimisation in a total birth cohort Am J Public Health 2005 95 2015 2021 10.2105/AJPH.2003.021436 16254233 PMC1449477

[B13] Silver E Piquero AR Jennings WG Piquero NL Leiber M Assessing the Violent Offending and Violent Victimisation Overlap Among Discharged Psychiatric Patients Law Hum Behav 2010 35 49 59 20145985 10.1007/s10979-009-9206-8

[B14] Chapple B Chant D Nolan P Cardy S Whiteford H McGrath J Correlates of victimisation amongst people with psychosis Soc Psychiatry Psychiatr Epidemiol 2004 39 836 840 15669665 10.1007/s00127-004-0819-4

[B15] Walsh E Moran P Scott C McKenzie K Burns T Creed F Murray RM Prevalence of violent victimisation in severe mental illness Br J Psychiatry 2003 183 233 238 10.1192/bjp.183.3.233 12948997

[B16] Teasdale B Mental disorder and violent victimisation Crim Justice Behav 2009 36 513 535 10.1177/0093854809331793

[B17] Hodgins S Lincoln T Mak T Experiences of victimisation and depression are associated with community functioning among men with schizophrenia Soc Psychiatry Psychiatr Epidemiol 2009 44 448 457 10.1007/s00127-008-0460-8 19030767

[B18] Choe JY Teplin LA Abram KM Perpetration of violence, violent victimisation, and severe mental illness: Balancing public health concerns Psychiatr Serv 2008 59 153 164 10.1176/appi.ps.59.2.153 18245157 PMC3756896

[B19] Hodgins S Alderton J Cree A Aboud A Mak T Aggressive behaviour, victimisation and crime among severely mentally ill patients requiring hospitalisation Br J Psychiatry 2007 191 343 350 10.1192/bjp.bp.106.06.029587 17906245

[B20] Dekker JJ Theunissen J Van R Peen J Duurkoop P Kikkert M Victimisation of patients with severe psychiatric disorders: prevalence, risk factors, protective factors and consequences for mental health. A longitudinal study BMC Public Health 2010 10 687 10.1186/1471-2458-10-687 21067566 PMC2991298

[B21] Dean K Moran P Fahy T Tyrer P Leese M Creed F Walsh E Predictors of violent victimisation amongst those with psychosis Acta Psychiatr Scand 2007 116 345 353 10.1111/j.1600-0447.2007.01078.x 17868429

[B22] Honkonen T Henriksson M Koivisto A-M Stengård E Salokangas RKR Violent victimisation in schizophrenia Soc Psychiatry Psychiatr Epidemiol 2004 39 606 612 15300370 10.1007/s00127-004-0805-x

[B23] Schomerus G Heider D Angermeyer MC Bebbington PE Azorin JM Brugha T Toumi M Urban residence, victimhood and the appraisal of personal safety in people with schizophrenia: results from the European Schizophrenia Cohort (EuroSC) Psychol Med 2008 38 591 597 17935638 10.1017/S0033291707001778

[B24] Silver E Mental disorder and violent victimisation: The mediating role of involvement in conflicted social relationships Criminology 2002 40 1 191 212 10.1111/j.1745-9125.2002.tb00954.x

[B25] Swanson JW Swartz MS Van Dorn RA Elbogen EB Wagner HR Rosenheck RA Lieberman JA A national study of violence behavior in persons with schizophrenia Arch Gen Psychiatry 2006 63 490 499 10.1001/archpsyc.63.5.490 16651506

[B26] Fitzgerald PB de Castella AR Filia KM Filia SL Benitez J Kulkarni J Victimisation of patients with schizophrenia and related disorders Aust N Z J Psychiatry 2005 39 169 174 10.1080/j.1440-1614.2005.01539.x 15701066

[B27] Goodman LA Salyers MP Mueser KT Rosenberg SD Swartz M Essock SM Swanson J Recent victimisation in women and men with severe mental illness: prevalence and correlates J Trauma Stress 2001 14 615 632 10.1023/A:1013026318450 11776413

[B28] Walsh E Gilvarry C Samele C Harvey K Manley C Tattan T Fahy T Predicting violence in schizophrenia: A prospective study Schizophr Res 2004 67 247 252 10.1016/S0920-9964(03)00091-4 14984884

[B29] Chen X The link between juvenile offending and victimisation Youth Violence and Juvenile Justice 2009 7 119 135

[B30] Brekke JS Prindle C Bae SW Long JD Risks for individuals with schizophrenia who are living in the community Psychiatr Serv 2001 52 1358 1366 10.1176/appi.ps.52.10.1358 11585953

[B31] American Psychiatric Association Diagnostic and statistical manual of mental disorders (Text revised, 4th ed.) 2000 American Psychiatric Press, Washington DC

[B32] Fellows L Castle D Ahmad F Validation and development of the use of the Very Brief Psychosis Treatment Schedule (VBPTS) Enhancing Ment Health Serv 2001 1 65 67

[B33] Kay SR Opler LA Fiszbein A The Positive and Negative Syndrome Scale (PANSS) Manual 2000 Multi-Health Systems Inc., Toronto

[B34] Skinner HA The drug abuse screening test Addict Behav 1982 7 363 371 10.1016/0306-4603(82)90005-3 7183189

[B35] Dyson V Appleby L Altman E Doot M Luchins DJ Delehant M Efficiency and validity of commonly used substance abuse screening instruments in public psychiatric patients J Addict Dis 1998 17 2 57 76 10.1300/J069v17n02_05 9567226

[B36] Yudko E Lozhkina O Fouts A A comprehensive review of the psychometric properties of the Drug Abuse Screening Test J Subst Abuse Treat 2007 32 2 189 198 10.1016/j.jsat.2006.08.002 17306727

[B37] Mossman D Assessing predictions of violence: Being accurate about accuracy J Consult Clin Psychol 1994 62 783 792 7962882 10.1037//0022-006x.62.4.783

[B38] Maniglio R Severe mental illness and criminal victimisation: a systematic review Acta Psychiatr Scand 2008 119 180 191 19016668 10.1111/j.1600-0447.2008.01300.x

[B39] Wallace C Mullen PE Burgess P Criminal offending in schizophrenia over a 25-year period marked by deinstitutionalization and increasing prevalence of comorbid substance use disorders Am J Psychiatry 2004 161 716 727 10.1176/appi.ajp.161.4.716 15056519

[B40] Arseneault L Moffitt TE Caspi A Taylor PJ Silva PA Mental disorders and violence in a total birth cohort: Results from the Dunedin study Arch Gen Psychiatry 2000 57 979 986 10.1001/archpsyc.57.10.979 11015816

